# Public health professionals delivering better health for all—50 years of the Faculty of Public Health

**DOI:** 10.1093/pubmed/fdac110

**Published:** 2022-11-21

**Authors:** Farhang Tahzib

**Affiliations:** Public health physician, Chair, Public health ethics committee, UK Faculty of Public Health

''**Wanderer, there is no path, the path is made by walking. Walking makes the path, and in glancing back one sees the path that will never be trod again**.'' Antonio Machedo (1912)^1^

The 50th Anniversary of the UK Faculty of Public Health (FPH) provides an opportunity to celebrate its achievements and reflect on its part in the remarkable history of public health and the public’s health in the UK and beyond. History is not merely a record of past events but the story of how purpose is accomplished and learnings in the advancement of the process towards a purpose, goal or mission and implications for the future. The creation of the FPH has been a significant milestone in the growth and development of public health as a professional discipline in the continuing efforts to promote public’s health and achieve greater social justice.

The FPH (formerly the Faculty of Community Medicine and then the Faculty of Public Health Medicine) was formed in 1972 as a result of a key recommendation of the Royal Commission on Medical Education (1965–68). It was set up as a joint, autonomous faculty by the three Royal Colleges of Physicians of the UK. At the inaugural meeting of the Faculty of Community Medicine on 15 March 1972, Lord Rosenheim, opened the meeting by noting that *‘this is a historic occasion’* and quoted from the Standing Orders that *‘Community Medicine is that branch of medicine which deals with populations or groups rather than individual patients’*, and thus, it ‘*brings together within one discipline those who are presently engaged in the practice of public health, in the administration of the health services whether in hospital, local authority or central government, in relevant research and those responsible for undergraduate and postgraduate education in the University Departments of Social medicine’.*^2^

The first Board of the Faculty included distinguished doctors, all white men apart from Dr Maud Perry Menzies, the distinguished community physician specializing in maternal and child health from Glasgow in Scotland. Fifty years on the FPH has worked to enable non-medical public health professionals become formally recognized as members of the Faculty, with a President from non-medical background, the Faculty has had a third of its Presidents as women, more than any other of the Royal Colleges, and now has its first Black President.

The FPH is the standard setting body for public health specialists within the UK, setting standards for training, examination and specialist practice across the four countries of the UK. It is also a source of knowledge and guidance around public health and advocates for public health nationally and globally. As the professional membership body for public health it works to promote and protect human health and its wider determinants for everyone in society by: *‘Playing a leading role in assuring an effective public health workforce; promoting public health knowledge and advocating for the reduction of inequalities and for the very best conditions for health and well-being to flourish’*.

## Marking 50 years of the FPH

The *Journal of Public Health* (JPH) was originally established in 1892 as the *Journal of State Medicine*, a journal for public health professionals. It has gone undergone several renames during its history from the *Journal of State Medicine*, *Journal of Preventative Medicine*, *Journal of Community Health*, *Journal of Community Medicine*, *Journal of Public Health Medicine* and since 2004 as the JPH, when non-medical public health professionals formally became members of the Faculty. It is befitting that the Journal is now marking the 50th anniversary of the FPH with a special supplement. The call for papers for the supplement provided opportunity for public health professionals, historians and scholars to reflect on the profession, the Faculty and the public’s health. This has resulted in a wide range of submissions on diverse issues and from diverse approaches, not only historical recordings, analysis and insights of activities of the past but also learnings and reflections on implications for the future.

A number of submissions have considered historical developments of the Faculty, its work and the times. This includes a paper by Samuel Trethway on the history of the FPH, considering the origins of public health in the UK and the role of the FPH in shaping public health practice and policy. Fiona Sim, Jenny Wright and Katie Ferguson robustly chronicle and analyse the development of a multidisciplinary public health workforce in the UK. This has been a significant 50-year journey, beginning at the time of the creation of the Faculty, which has shaped public health specialist practice, transforming the FPH identity and membership and an opportunity for leadership. Frank Houghton, in a commentary on a multidisciplinary public health workforce, argues that although much has been done, there is a need to do more, particularly around sharing learning and influencing other public health systems around the world to recognize the importance of a multidisciplinary public health workforce. A short paper by Paul Johnstone, shares his reflection on the development of the Faculty International Committee and the importance of global health issues to the Faculty and its work.

Jenny Wright, in a scholarly paper on ‘Public health women doctors in England: from backwater to strategic roles in 20 years’, provides a detailed account of the history of public health medicine in England between 1974 and 1990s. She describes how, through a combination of design and happenstance in response to organizational changes, a gender-neutral speciality was created, and women doctors in public health were able to achieve consultant status, and major strategic leadership roles, well ahead of female colleagues in other areas of medical practice. Public health professionals work across many sectors. The paper by Major Falconer-Hall, Lieutenant General Professor Bricknell and Colonel Ross highlight the role of public health specialists within the UK Defence Medica Services. They note that the first professor of military hygiene, Dr Edmund Parkes, was a leading pioneer in the public health movement of the late 19th century, and how since then, the armed forces have evolved from the term ‘hygiene’, through to ‘health’ to ‘well-being’.

Alex Mold, Director of the Centre for History in Public Health at the London School of Hygiene and Tropical Medicine (LSHTM), explores a set of changes and continuities in relation to public health and its publics in the UK since the establishment of the FPH. She identifies three key issues in her commentary: who has responsibility for public health; the persistence of social and racial inequalities in population health and the ‘return’ of infectious disease as a threat to public health. She concludes that despite the trend to place more responsibility for individual and collective health on the public itself, there has been a proliferation in the actors and authorities involved in securing and protecting the health of the public. The strong linkages between health and structural inequality, and the challenges of addressing these, demonstrate that public health never was (and never can be) solely an individual matter.

It has been surprising that despite efforts to include them, there were no submissions on the public health curriculum, examinations, accreditation and related activities. Such activities are often taken for granted, done routinely and occur behind the more visible work around advocacy, policy development and leadership to support the public health workforce. Yet education and training are at the heart of the work of the Faculty and it has been leading the field globally, as recognized, for instance, when it was awarded the ASPHER Deans and Directors Good Practice Award for excellence in public health training and education in 2021.

Many of the authors and submissions have been particularly interested in who we are, where we have come from, how we are doing and how we should work through the organized efforts of society to promote and protect public’s health and social justice. They offer challenges to the FPH and other public health leaders that we need to reflect on; to learn from the past and reflect more deeply on who we are, where we are going and consider new ways of thinking and acting if we are to meet the remarkable public health challenges of the next 50 years.

## “If we could first know where we are, and whither we are tending, we could then better judge what to do, and how to do it.” Abraham Lincoln (1858)

The 1920 classic definition of public health by Charles-Edward Winslow as *‘the science and art of preventing disease, prolonging life, and protecting physical health and efficiency through organised community efforts,’* continues to be broadly used. The definition by Winslow appeared in the journal *Science* in his paper, the *‘untilled fields of public health*’,^3^ sharing the reflections he had with two undergraduate medical students seeking his advice around choice of a career in particular in the field of public health and the opportunities and tendencies of modern public health practice at the time. He concluded his paper by citing Sir John Simon, the first Chief Medical Officer for England and Wales in1855, that, ‘*The canker of …. disease gnaws at the very root of our national strength. The sufferers are not few or insignificant. They are bread winners for at least a third part of our population…. That they have causes of disease indolently left to blight them amid their toil …. Is surely an intolerable wrong. And to be able to redress that wrong is perhaps among the greatest opportunities for good which human institutions can afford’.* Winslow believed that equal in weight with scientific ideas about health and disease was a commitment to social justice—that social ills must be *‘the first conquest in the conquest of epidemic disease’*. The values and the moral mission of public health are indeed what define public health professionals and are what have drawn many people into the profession.^4^

In the UK, the contemporary speciality of public health has its origins rooted in Victorian England, with ‘the Great Sanitary Awakening’ of the 19th century acting as key drivers of social reform and resulting in paradigm shift in how society viewed health and illness. The work by Edwin Chadwick, public health leaders and reformers at the time forced a nation into awareness of its responsibilities in regard to health, highlighting the links between poverty and wider determinants of health. The landmark legislation passed around water supply, sewage and sanitation in the 19th century in the UK were, as Lord Campbell noted in Parliament at the time, *‘the very first time that the legislature of any country had proposed to frame a general measure for improving and securing the general health of the community’*. The 1848 Public Health Act signified a major turning point in the UK public health landscape leading to formation of the ‘General Board of Health’, local ‘Boards of Health’ and widespread employment of ‘Medical Officers of Health’ within local authorities and appointment of a Chief Medical Officer in 1855 charged with the responsibility of overseeing the health of the population of the Country. These changes laid the foundations for modern public health practice in the UK.

Prior to the establishment of the Faculty, the training for public health and the statutory qualifications, the DPH, was provided by a number of bodies. But the courses were variable, not stimulating, concentrating on laws and regulations with very little on epidemiology, non-communicable diseases and other key issues. The academic base of public health was also insecure, with for example no academic departments of public health in any of the 12 London medical schools. Therefore, the creation of the UK FPH has been a key part of the agenda in the professionalization of public health, providing standards, education, training and support for the professional public health workforce and leadership and advocacy for the public’s health.

The paper by Katarzyna Czabanowaska and John Middleton, former Presidents of ASPHER and the FPH, on Professionalism of the public health workforce, highlights the need and case for a skilled, expert, recognized and regulated public health profession to protect and improve the health of people and the planet. They note that the work of the FPH and the UK system ‘*is widely admired but rarely emulated’*. This they suggest is *‘because of the absence of that professional recognition, the regulatory bodies and self-governing of standards that the UK Faculty takes forward’*. Two commentaries on their paper from scholars in the USA, where the American Public Health Association are celebrating their 150th anniversary also this year, suggest that there is more to do and that in the US *‘the public health workforce needs a revolution, starting with unification, to achieve the power it needs to protect the public’s health’,* and that this is ‘*a time for professionalism, standard setting, and investment in the workforce’.*

## Back to our roots or sowing new seeds?

Walter Holland, member of the provisional and the first Board of the Faculty and a distinguished scholar, in his personal account of the Creation of the FPH on the FPH website^5^ notes that, *‘looking back on the foundations of the Faculty it is important to be aware that Public Health, after its 19th century achievements, has always had difficulties in establishing its role and esteem. The dramatic advances in treatment first of infective conditions and later of chronic conditions such as coronary heart disease have always, in the public mind, overshadowed the far more effective public health measures such as vaccination, or the identification of hazards of smoking and its prevention, lack of exercise and diet in the control of disease’*. There have perhaps also been issues around public health professionals themselves not being comfortable and appreciating their role in advocacy and their professional mandate and responsibility in social reform. The professionalization of higher education has perhaps siloed and splintered off public health from social work, sociology, ethics, law and other disciplines. There is clearly a need to build capacity and competence of the workforce around such issues, and greater understanding around the public health mandate, its foundational core values and their implications for policy-making and day-to-day practice.

May Van Schalkwyk and fellow researchers at the Commercial Determinants Research Group at LSHTM, in their paper, call for *‘thinking anew on the paradigms of health, harm and disease’*. They lament that the popular narrative on health is still dominated by a biological model that focuses on disease-causing pathogen or agent that leads to pathology, which is diagnosable and amenable to intervention at the individual level via measures delivered through the healthcare and public health systems, and challenge our ways of thinking about health, harms and disease. Silvio Brusaferro, Laura Brunelli and group of public health academics from Italy in their paper argue that there is need to review and update the traditional public health domains from the traditional 3 Ps to 6Ps. That is to add Prediction, Precision and Participation to the current accepted public health domains of health Promotion, Prevention and Protection.

## Reclaiming the public health mandate

The image of public health professionals has ebbed and flowed historically. Public health and its practitioners were not held in high esteem by the medical professions at the beginning of the 20th Century, often referred to as the *‘drains doctors’*, and subsequently as bureaucrats as characterized by Dr Snoddie in Dr Finlay’s Casebook classic TV series in 1960s. *‘Fiction, never kind to public health officers’ *according to Rosalind Stanwell-Smith and Deidre Hine in 2001, *‘still portrays the service practitioners as a motley crew of generally negative officials’*, and that *‘the stereotype of unhelpful management lackey persists’.*^6^ There is a general image of public health practitioners as workers in a wide mix of poorly understood specialised areas; conversations at the dinner table around what you do for work perhaps take longer to explain than they do for members of other professions.

Ed Young, who won the Pulitzer Prize for Explanatory Reporting for his coverage of the COVID-19 pandemic, in a piece *‘How public health took part in its own downfall—the field’s future lies in reclaiming parts of its past that it willingly abandoned’*,^7^ published in the Atlantic, noted that public health *‘in the 19th century was not a narrow scientific endeavour but one that stretched across much of society. Those same broad networks and wide ambitions’ *he suggests, *‘are necessary now to deal with the problems that truly define the public’s health’*. Amy Fairchild and colleagues in their work on ‘the exodus of public health, what history can tell us about the future’^8^ concluded that: ‘*If a commandment emerges from history, it is one that all sectors can heed: find ways to align with constituencies, lend our science and our knowledge, and create a base of power for progressive social change’*.

There are a number of issues that have become clear, and better understood, with the COVID-19 pandemic and the prevailing public health challenges. First, the importance of protecting and promoting the public’s health; second, the fundamental need and value of public health professionals; and third, the need for public health professionals to be well trained, resilient and be steadfast to their professional public health mandate.

At the start of the pandemic in 2020 people went onto their balconies and front doorsteps to applaud and convey their admiration and thanks to health care and public health workers for their service and expertise. But by 2021 there were reports of public health practitioners being abused, at times even their lives threatened and attacked for simply trying to do their jobs in devising and advocating policies around vaccination, masks and other public health interventions to protect and promote the health of the people who they were serving. Nanny state slurs and accusations continue to be regularly used to attack health groups and governments, as cliches and slogans to discredit us while avoiding discussion, debate and facts. There is evidence of growing moral distress and injury of the UK public health workforce. There is nothing new about demonising and slogans in opposing public health measures. In 1854, when Edwin Chadwick pressed for basic sanitation, the London Times noted that, *‘we prefer to take our chances of cholera and the rest than be bullied into health by Mr Chadwick’.*^9^ As highlighted by Daube, ‘there are legitimate debates to be had about legislation, taxation, public education and other approaches to protecting the public’s health. But they should focus on the issues, not on slogans and cliches’^10^ and political ideologies.

Michael Marmot a distinguished, internationally recognized scholar and Fellow of the FPH started his pioneering, influential work on social determinants of health in 1972, the same year as the FPH was created. In his commentary for the supplement he provides personal reflections and powerful insights around his significant work and involvement as a public health professional for the past half century to promote health equity and create a better society. He reiterates his powerful Declaration from the WHO Commission on Social Determinants of Health, which he chaired, that *‘social injustice is killing people on a grand scale’* and invites all those working to promote health equity; in the words of Chilean poet Pablo Neruda, to *‘Rise up with me against the organisation of misery’*.

## Is public health just science?

Michael Marmot notes that *‘action to achieve greater health equity cannot avoid politics. And, if indeed such actions are a key part of public health, public health has to confront politics’*. Martin McKee, a prolific scholar, and former President of the European Public Health Association, in his provocative paper for the supplement argues that politics is at the heart of public health and the key question is whether we are willing to engage. He reflects on the challenges to governance in the UK outside of the European Union. Drawing on Virchow’s call to address the political determinants of health, he is critical of the current government and argues that it undermines the trust that is essential for effective public health policies that attract public support. He directly challenges the public health establishment in the UK to decide whether they will challenge the decline in public standards, and thus promote structures that can consider and prioritize health, or continue to pick up the broken pieces of political failings.

John Coggon, a respected public health ethico-legal scholar and thinker, in his paper for the supplement, poses a question as to whether public health is just science. He highlights the essentially political nature of public health as well as reasons why members of the public health community might aim not to be political in their practice. He notes there are potentially three issues to be considered: professional competence/expertise; democratic legitimacy and possibly damaging consequences of being seen as engaged in politics. Such considerations, he suggests, while valid, do not stop public health from being political in nature and he explores ideas around approaches to political engagement, asking how and when members of the public health community and its institutions, such as the FPH, might or might not seek to realise the moral mandate of public health in their practice through engagement with politics.

## Protagonist for health and social justice

The FPH in the past 50 years, through the continuous restructuring of the NHS and the political climates and flavours of the times, has had to be a vigilant advocate for the public’s health and a public health system to meet the public’s health needs and challenges, and actively support the public health workforce through uncertainty and change. With continuing and growing pressures and threats to public health budgets, public health’s independence and the fragmentation of the workforce there has been a tendency to consider public health as merely a diverse set of technical specialties serving organizational and corporate outcomes. Yet many public health professionals argue that, *‘values define us as a group of public health professionals and values drew many of us into public health’* and *‘that fundamental ethical and basic scientific values support the mission and purpose of the profession*’.^4^ There has been a call to train and support the next generation of public health leaders to have fire in their bellies, to be heroes for protecting and promoting the public’s health and social justice and to move from medical interventionist approaches towards a values-driven approach based on courageousleadership and advocacy.

There has been a tendency to create false dichotomies in considering the individual versus the collective or the institutions of society as mutually exclusive alternative choices in promoting and protecting health and social justice. The idea of a divide between, on the one hand, individual freedoms and personal choice and, on the other hand, the state and authority demanding submission has led to polarization, conflict and continuance of outdated ideologies and approaches to achieving the potential of organized efforts of society. Yet there is growing recognition that the individual, the community and the institutions of society, which serve us are all key protagonists and chief stewards for health, from whose mutually enriching values and interactions the future emerges. It is through understanding the interdependence, interactions and relationships between these key protagonists that we can create healthy communities, served by just institutions, in which individuals and families can flourish.

The FPH will be facing significant public health challenges in the next 50 years. These will be crucial, defining years for the planet, its people and their health. How we work to support efforts to tackle climate change, global emergencies, other public health challenges and the establishment of a new world order and systems to promote equity, social justice and meet the exigencies of our times will be critical. It would be useful to remind ourselves of the words of the British physician, Dr Elizabeth Blackwell (1821–1910), the first woman on the Medical Register of the General Medial Council in the UK that: *‘We are not tinkers who merely patch and mend what is broken’ *but rather, *‘we must be watchmen, guardians of the life and the health of our generation, so that stronger and more able generations may come after’.*

## References

[1] ''Proverbios y cantares XXIX'' [Proverbs and Songs 29], Campos de Castilla (1912); trans . In: Craige BJ (ed). Selected Poems of Antonio Machado. USA: Louisiana State University Press, 1979.

[2] Warren MD . The creation of the Faculty of Community Medicine (now the Faculty of Public Health Medicine) of the Royal Colleges of Physicians ofthe United Kingdom. J Public Health 1997;19(1):93–105. 10.1093/oxfordjournals.pubmed.a024596 9138225

[3] Winslow CE . The untilled fields of public health. Science 1920;51(1306):23–33. 17838891 10.1126/science.51.1306.23

[4] Coughlin SS . Ethics in epidemiology at the end of the 20th century: ethics, values, and mission statements. Epidemiol Rev 2000;22:169–75. 10939024 10.1093/oxfordjournals.epirev.a018016

[5] Holland W . The creation of the Faculty of public health. A Personal Reflection. Walter Holland, https://www.fph.org.uk/media/1382/the-creation-of-the-faculty-of-public-health-walter-holland.pdf.

[6] Stanwell-Smith R , Hine D. Public health medicine in transition. J R Soc Med 2001;94(7):319–21. 11418699 10.1177/014107680109400701 PMC1281593

[7] Yong ED. How public health took part in its own downfall. The Atlantic. 2021. https://www.theatlantic.com/health/archive/2021/10/how-public-health-took-part-its-own-downfall/620457/.

[8] Fairchild AL , Rosner D, Colgrove J et al. The exodus of public health what history can tell us about the future. Am J Public Health 2010;100(1):54–63. 19965565 10.2105/AJPH.2009.163956 PMC2791244

[9] Ferriman A . Vilified for tackling tobacco. BMJ 2000;320(7247):1482. 10827068 PMC1127662

[10] Daube M , Stafford J, Bond L. No need for nanny. Tob Control 2008;17:426–7. 19029365 10.1136/tc.2008.027763

