# Correction to: Public health professionals delivering better health for all—50 years of the Faculty of Public Health

**DOI:** 10.1093/pubmed/fdad021

**Published:** 2023-03-31

This is a correction to: Public health professionals delivering better health for all—50 years of the Faculty of Public Health, *Journal of Public Health*, Volume 44, Issue Supplement_1, November 2022, Pages i3–i7, https://doi.org/10.1093/pubmed/fdac110

In the originally published version of this manuscript the author Farhang Tahzib was inadvertently omitted from the author byline. The publisher apologizes to the author for this omission.

This error has been corrected online.

